# Natural Cinnamaldehyde and Its Derivatives Ameliorate Neuroinflammatory Pathways in Neurodegenerative Diseases

**DOI:** 10.1155/2020/1034325

**Published:** 2020-11-14

**Authors:** Mehrdad Hajinejad, Maryam Ghaddaripouri, Maryam Dabzadeh, Fatemeh Forouzanfar, Sajad Sahab-Negah

**Affiliations:** ^1^Student Research Committee, Mashhad University of Medical Sciences, Mashhad, Iran; ^2^Neuroscience Research Center, Mashhad University of Medical Sciences, Mashhad, Iran; ^3^Shefa Neuroscience Research Center, Khatam Alanbia Hospital, Tehran, Iran; ^4^Department of Neuroscience, Faculty of Medicine, Mashhad University of Medical Sciences, Mashhad, Iran

## Abstract

Neurodegenerative diseases are devastating and incurable disorders characterized by neuronal dysfunction. The major focus of experimental and clinical studies are conducted on the effects of natural products and their active components on neurodegenerative diseases. This review will discuss an herbal constituent known as cinnamaldehyde (CA) with the neuroprotective potential to treat neurodegenerative disorders, such as Alzheimer's disease (AD) and Parkinson's disease (PD). Accumulating evidence supports the notion that CA displays neuroprotective effects in AD and PD animal models by modulating neuroinflammation, suppressing oxidative stress, and improving the synaptic connection. CA exerts these effects through its action on multiple signaling pathways, including TLR4/NF-*κ*B, NLRP3, ERK1/2-MEK, NO, and Nrf2 pathways. To summarize, CA and its derivatives have been shown to improve pathological changes in AD and PD animal models, which may provide a new therapeutic option for neurodegenerative interventions. To this end, further experimental and clinical studies are required to prove the neuroprotective effects of CA and its derivatives.

## 1. Introduction

Neuroinflammation plays a critical role in the pathogenesis of both acute and chronic neurological diseases, exemplified by stroke, Alzheimer's disease (AD), Parkinson disease (PD), and depression. During the neuroinflammatory process, immune and glial cells are extremely activated and release several proinflammatory mediators that cause neural injury. Thus, treatment or prevention of neuroinflammation-mediated neural tissue dysfunction may have a potential therapeutic option against neurological diseases [1, 2].

Herbal drugs, such as plant, spices, and their oils, have been used from ancient times to today to treat neurological disorders [3]. It has been shown that herbal drugs exert anti-inflammatory properties in a variety of peripheral systems. Now, increasing evidence indicates that anti-inflammatory herbal medicine and its constituents are being verified to be a potent neuroprotector against numerous brain disorders [4–6].

**Figure 1:**
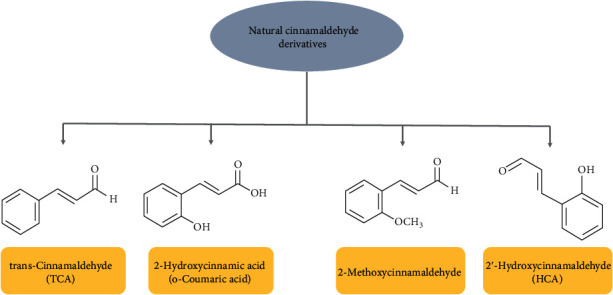
Chemical structures of natural cinnamaldehyde derivatives.

**Figure 2:**
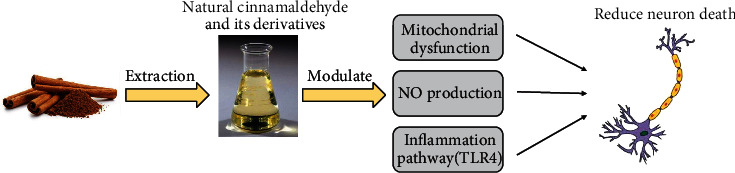
Schematic diagram of cinnamaldehyde extraction and its ability to reduce neuronal cell death through the inhibition of mitochondrial dysfunction, nitrite oxide (NO) production, and inflammatory pathways.

Cinnamon powder is one of the most famous spices made from the inner bark of cinnamon trees. The most common species are *C. cassia* (Chinese cinnamon) and *C. verum* (true cinnamon). These two species contain 85.3 and 90.5 percent of the cinnamaldehyde (CA) [7]. CA is a major bioactive of bark extract of cinnamon that is responsible for the odor and flavor of cinnamon [8]. This component was isolated from cinnamon for the first time by Jean-Baptiste Dumas and Eugène-Melchior Péligot in 1834 and then synthesized in the laboratory by the Italian chemist, Luigi Chiozza in 1854 [9, 10]. Thus, CA can be obtained from natural extracts or synthesized in a laboratory. Natural CA derivatives include *trans*-cinnamaldehyde (TCA), 2′-hydroxycinnamaldehyde (HCA), 2-methoxycinnamaldehyde (MCA), and 2-hydroxycinnamic (o-Coumaric acid) (Figure 1) [11]. CA derivatives are structurally identified by the presence of cinnamoyl moiety [12]. In their structures, the presence of highly reactive *α*,*α*-unsaturated carbonyl pharmacophore (Michael acceptor) can react with some enzymes and/or receptors as electrophiles and subsequently generate different therapeutically relevant pharmacological functions [12]. Natural CA and its derivatives have been extensively investigated and comprehensively reviewed with a wide range of effects, such as anticancer, antidiabetic, antifungal, and antibacterial [7, 11, 13, 14]. Besides, natural CA and its derivates have been used for neurological purposes because they have anti-inflammatory, neuroprotective, antioxidative stress, and antiapoptotic properties (Table 1) and (Figure 2) [15, 16]. For example, CA exhibits a protective influence against glutamate-induced cytotoxicity in PC12 cells [17]. CA considerably attenuated cell survival, inhibited the generation of reactive oxygen species (ROS), alleviated mitochondrial membrane potential, reduced the production of cytochrome c, and decreased the activities of caspase-3 [18]. CA also significantly enhanced Bcl-2 (as an antiapoptotic marker) while reducing Bax (as a proapoptotic marker) expression [17]. Moreover, TCA as a main natural CA derivative significantly decreased LPS-induced neuronal death through the inhibition of neuroinflammation by blocking the nuclear factor kappa B (NF-*κ*B) signaling pathway in vitro condition [19]. In the same way, HCA is another CA derivative that can inhibit neuroinflammatory signaling pathways such as NF-*κ*B, extracellular-regulated kinase (ERK), c-Jun N-terminal kinase (JNK), and p38 mitogen-activated protein kinase (p-38 MAPK) by targeting low-density lipoprotein receptor-related protein 1 (LRP1). [20]. Therefore, natural CA and its derivatives may be used as a therapeutic agent against neuroinflammation for improving neurological disorders. However, the mechanisms of natural CA and its derivatives on neuroinflammation should be studied more than that it has. To this end, the aim of this study was to review the current status of the neuroprotective and antineuroinflammatory properties of natural CA and its derivatives and discuss their potentials as therapeutic agents in neurodegenerative diseases.

## 2. Natural CA and Its Derivatives Act as a Neuroprotective Agent by Targeting Neuroinflammatory Pathways

**Figure 3:**
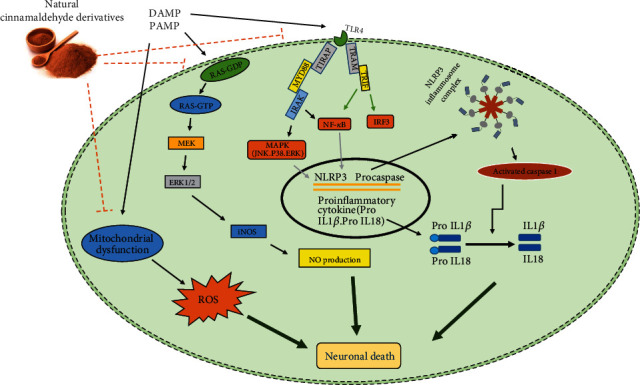
Neuroprotective effects of cinnamaldehyde on neuroinflammation, oxidative stress, and mitochondrial dysfunction. Abbreviation: PAMP: pathogen-associated molecular pattern; DAMP: damage-associated molecular pattern.

Neuroinflammation is an intrinsic brain response that involves neurons and glial cells within the central nervous system. The neuroinflammation signaling pathways are the subject of extensive experimental and clinical studies [21–23]. The NF-*κ*B pathway is a main proinflammatory cytokine mediator that is activated by toll-like receptors (TLRs) [24]. The TLRs are the most prominent family of pattern recognition receptors that are a part of the innate immune response [25, 26]. Stimulation of TLRs by insult factors leads to severe inflammatory responses by releasing proinflammatory cytokines, such as interleukin-1*β* (IL-1*β*) and interleukin-18 (IL-18) [27]. TLR4 is a membrane receptor of TLRs which have two adaptor proteins including myeloid differentiation primary response gene 88 (MyD88-) dependent pathway and MyD88-independent pathway (TRIF-dependent pathway) [28]. Activation of TLR4 recruits MyD88 and activates NF-*κ*B that consequently expresses proinflammatory cytokines [29]. Considerable data have shown that the TLR4/NF-*κ*B signaling pathway plays a key role in the pathogenesis of neuroinflammation [30]. Therefore, attenuating the TLR4/NF-*κ*B signaling pathway can be considered as a therapeutic strategy for treating brain diseases [31]. With this in mind, it has been revealed that CA inhibited TLR4/NF-*κ*B signaling and NLRP3 (caspase-1-containing multiprotein complex) inflammasome and subsequently controlled the release of IL-1*β* and IL-18 during the inflammatory processes [32]. Furthermore, CA suppresses NLRP3 inflammasome activation via inhibiting the cathepsin B and P2X7R ((P2 receptors) protein expression [33]. In this case, TCA improves depressive-like behaviors in a chronic mild stress model by inhibiting the NF-*κ*B/NLRP3 inflammasome pathway [33]. Also, CA not only inhibits TLR4/NF-*κ*B signaling but also attenuates the increased levels of TNF-*α*, IL-1*β*, C-C Motif Chemokine Ligand 2 (CCL2), and endothelial-leukocyte adhesion molecule-1 (ELAM-1) in a cerebral ischemic model that ultimately decreased leukocyte infiltration into the ischemic brain areas (Figure 3) [32]. CA also suppressed cytokine secretion from lipopolysaccharide (LPS-) activated macrophages [34]. Additionally, CA inhibited apoptosis and ROS generation by inhibiting NF-*κ*B activity in the DRG neurons treated by high glucose as an in vitro neuropathy model [35].

The molecular mechanism of CA and its derivatives are not fully understood, but these results suggest that natural CA and its derivatives may introduce as a new candidate for further development as an anti-inflammatory agent for neurodegenerative diseases [34, 36–40].

## 3. CA Attenuates the Progression of Neurodegenerative Diseases

### 3.1. Alzheimer's Disease

AD is known as a progressive age-related neurodegenerative disorder. The symptoms of AD depend on the stage of the disease that is classified into early-stage, mild, moderate, and late-stage according to the degree of cognitive impairment. AD is associated with neurological and motor dysfunction that ultimately results in progressive memory loss [41–43]. The exact cause of AD is not well understood, but oxidative damage and excessive ROS production have been characterized at the early stage of AD. Moreover, the main hallmark pathology of AD is the accumulation of amyloid plaques and hyperphosphorylated Tau proteins in the brain that these events cause neuronal cell death through a series of toxic pathways [44, 45]. Despite huge basic and clinical research on AD, there is no effective treatment to stop the progression of clinical symptoms in AD. Additionally, other pathological features such as neuroinflammation, microglial activation, acetylcholinesterase (AChE) dysregulation, synaptic impairment, and mitochondrial dysfunction play important roles in the pathogenesis of AD. Therefore, it is necessary to develop multifunctional drugs with fewer side effects to target different aspects of AD pathology [46–48]. In this case, it has been reported that CA prevents the accumulation and formation of plaques and neurofibrillary tangles in neurons. CA also reduces the accumulation of Tau protein through interaction with the two cysteine residues in tau and prevents neuronal loss (Table 2) [49]. CA extract and its polyphenolic derivatives maintain redox homeostasis through free radical scavenging activities. It has been reported that long time consumption of cinnamon downregulated oxidative stress markers in the blood [50]. Besides, CA significantly improved the lifespan and healthspan in male AD flies [44]. Furthermore, it has been reported that the administration of CA (40 mg/kg) improved cognitive performance by increasing phosphorylated ERK1/2 in the prefrontal cortex of rats in the methamphetamine cognitive impairment model [51]. In addition, it has been shown that TCA possesses an ability to inhibit neuroinflammatory responses by declining the microglial activation and levels of proinflammatory mediators in the mice brain of the AD model [52]. Besides, TCA improves memory impairment by suppressing microglial activation [53]. For this reason, TCA considerably reduced nitric oxide (NO) production in microglial cells by accelerating the destabilization of inducible nitric oxide synthase (iNOS) mRNA through the disruption of the mitogen-activated protein kinase kinase (MEK1/2)-ERK1/2 pathway in a mouse memory impairment model of LPS [46]. TCA can also inhibit the NF-*κ*B pathway through the downregulation of iNOS, Cyclo-oxygenase-2 (COX-2), and TNF-*α* gene expressions in LPS-induced microglial cells [53]. Similarly, TCA can decrease the iNOS levels and phosphorylated ERK1/2 in the hippocampal tissue of the LPS in vivo model [46]. Recently, it has been reported that TCA has a memory-enhancing effect [54]. TCA improved the spatial memory and locomotor activity in mice with lipopolysaccharide (LPS-) induced memory impairment by stimulating the nuclear factor erythroid 2-related factor 2 (Nrf2) and restoring superoxide dismutase and glutathione-S-transferase as the downstream antioxidant enzymes in the hippocampus. TCA also decreased the levels of IL-1*β* and caspase-3 as well as A*β*1–42 protein accumulation in the brain of mice [55]. Therefore, TCA enhanced memory function through the amelioration of Nrf2, inhibition of neuroinflammation and apoptosis, and reduction of amyloid protein aggregation.

Another significant aspect of AD pathology is decreased synaptic protein expression and synaptic impairment. TCA can increase synaptic markers in the hippocampus and frontal cortex of the mouse model of neurological disorder, indicating an improvement in synaptic connection in AD [52, 56]. Besides, CA polyphenolics may improve dementia through their vasorelaxant potentials and attenuating vascular cell adhesion molecule expression within the endothelial cells [43]. These examples show that natural CA and its derivatives can improve AD symptoms by attenuating different pathological pathways.

### 3.2. Parkinson's Disease

PD is a debilitating progressive neurodegenerative disorder characterized by degeneration and loss of dopaminergic neurons in the substantia nigra area of the midbrain [54]. Mitochondrial dysfunction, neuroinflammation, oxidative stress, loss of supportive molecules, and dysregulated kinase signaling are critical factors that are known to impact the pathogenesis of PD. Moreover, preclinical and clinical evidence suggests that *α*-synuclein misfolding (Lewy body) and autophagy imbalance have been reported as the molecular mechanisms underlying PD pathogenesis [57, 58]. To date, there is no effective treatment to reduce the progression of PD or even to prevent its manifestation. Therefore, searching for neuroprotective agents, which can stop the underlying pathological condition and prevent further neuronal death, is needed. Among different neuroprotective agents, herbal components have long-term efficacy and safety [59, 60].

Studies have been demonstrated that cinnamon has anti-inflammatory effects as well as some neuroprotective properties [61, 62]. Treatment with cinnamon prevented the development of PD-like symptoms and pathology in 1-methyl-4-phenyl-1,2,3,6-tetrahydropyridine (MPTP-) treated mice [63]. The major compound in cinnamon is CA, which is metabolized into sodium benzoate (NaB) in the liver. NaB can cross the blood–brain barrier and increase the production of neurotrophic factors in the brain. It has been reported that NaB metabolized from cinnamon can inhibit the loss of Parkin and DJ-1 (protein deglycase), regulate neurotransmitter levels, and improve motor functions in mice with PD [63–65]. Parkin and DJ-1 are necessary proteins for supporting the survival of dopaminergic neurons while significantly decrease in the brain of PD patients [66]. Consequently, sodium benzoate (NaB) metabolized from cinnamon modifies the pathology of PD through the production of neurotrophic factors and inhibition of neuroinflammation [64]. NaB produces brain-derived neurotrophic factor and neurotrophin-3 *via* the activation of protein kinase A and cAMP response element-binding pathway [64]. On the other hand, NaB suppresses the activation of p21^ras^, a small G protein, and consequently decreases the activation of NF-*κ*B. Following the inhibition of NF-*κ*B activation, iNOS expression and NO production are reduced, which ultimately inhibits the neuroinflammation in PD [63, 67]. Furthermore, the inhibition of LPS-induced production of NO and expression of iNOS, COX-2, and IL-1*β* is the molecular mechanism behind TCA-mediated neuroprotective effects [19]. CA also exerted antioxidant activity by decreasing ROS production in 6-hydroxydopamine-induced cytotoxicity (Table 1) [68]. Recently, it has been shown that CA could block dysregulated autophagy in MPTP as a PD model [69]. In general, it seems that CA has a neuroprotective effect in PD models and might be a promising therapeutic target for PD.

## 4. Conclusion Remarks and Future Perspectives

The current treatment for neurodegenerative diseases is based on synthetic drugs that show undesirable side effects or toxicity. On the other hand, natural components are thought to be relatively safe and effective; therefore, many researchers have focused to find herbal products with neuroprotective effects. Initial experimental studies have shown that natural CA and its derivatives have therapeutic effects via modulation of several mechanisms, such as inflammation, mitochondrial dysfunction, and synaptic connection in neurodegenerative diseases, such as AD and PD. However, further studies in animal models and clinical trials are needed to clarify the safety and efficacy of CA and its derivatives as a potential therapeutic option for neurodegenerative disorders.

## Figures and Tables

**Table 1:** Experimental studies of natural cinnamaldehyde and its derivation on neurological diseases.

Natural cinnamaldehyde derivation	Model	Animal/cell	Effective dose	Mechanism	Result	Main effect	Ref.
CA	Permanent cerebral ischemia	Mouse	50 mg/kg	Suppress the activation of TLR4, TRAF6, and NF-*κ*B signaling	Attenuate levels of TNF-*α*, IL-1*β*, CCL2, and ELAM-1	Anti-inflammatory effect	[31]
CA	Glutamate toxicity	PC12 cells	20 *μ*M	Inhibit intracellular ROS accumulation, increase Bcl-2 expression, inhibit Bax expression, block the release of cytochrome c, and decrease the LC3-II/LC3-I ratio	Maintain normal mitochondrial membrane potential and prevent the activation of autophagy	Antioxidative stress and antiapoptotic effects	[17]
CA	Diabetic neuropathy	DRG	100 nmol/L	Inhibits the activation of NF-*κ*B pathway	Attenuate caspase-3 activation and downregulate IL-6, TNF-*α*, COX, and iNOS levels	Anti-inflammatory effect	[34]
CA	Sciatic nerve crush	Rat	30 mg/kg/day	Upregulate the number of regenerated nerves and Schwann cells	Promote nerve regeneration, motor function recovery, and muscle mass	Neuroprotective effect	[38]
TCA	Depression	Mouse	50 mg/kg	−	Regulate the level of GABA neurotransmitter and also regulate the eCB system by downregulation of TRPV1 and CB1	Antidepressive effect	[39]
HCA	LPS-induced neuroinflammation	BV-2 microglial cells	2 *μ*M	Inhibit ERK, JNK, p38 MAPK, and NF-*κ*B activation by target LRP1 and reduce microglia-mediated neuroblastoma cell death	Attenuate the expression of iNOS and proinflammatory cytokines such as IL-1*β* and TNF-*α*	Anti-inflammatory and neuroprotective effects	[19]

Abbreviations: HCA: 2-hydroxycinnamaldehyde; MCA: 2-methoxycinnamaldehyde; DRG: dorsal root ganglion neurons; LPS: lipopolysaccharide; TRAF6: tumor necrosis receptor-associated factor 6; TLR4: Toll-like receptor 4; eCB: endocannabinoid; MAPKs: mitogen-activated protein kinases, ERK: extracellular-regulated kinase; JNK: c-Jun N-terminal kinase; COX: cyclooxygenase; iNOS: inducible nitric oxide synthase; NO: nitric oxide; IL-1*β*: interleukin-1*β*; IL-6: interleukin-6; TNF-*α*: tumor necrosis factor-*α*; NF-*κ*B: nuclear factor kappa B; TRPA1: transient receptor potential ankyrin 1; ROS: reactive oxygen species; MDA: malondialdehyde; Bcl-2: B cell lymphoma 2; BAX: BCL2-Associated X; CCL2: C-C Motif Chemokine Ligand 2; ELAM-1: endothelial-leukocyte adhesion molecule 1; GABA: Gamma-Aminobutyric acid; TRPV1: transient receptor potential cation channel subfamily V member 1; LRP1: low-density lipoprotein receptor-related protein 1; LC3: microtubule-associated protein light chain 3.

**Table 2:** Preclinical studies of natural cinnamaldehyde and its derivation-mediated neuroprotective effects on Alzheimer's disease and Parkinson's disease models.

Model	Animal/cell type	Injection route	Duration	Compound	Effective dose	Mechanism	Main finding	Behavioral assessment	Ref.
Drosophila melanogaster (overexpressing A*β* or Tau proteins)	Fly	CA added to yeast paste	100 days	CA	80 mM	Activate the autophagy pathway by Nrf2	Improve the lifespan and healthspan of male AD flies	Improve climbing ability and improve short-term memory	[43]
Tau 187	_	Incubation	17 h	CA and epicatechin	110 *μ*M	−	Reduce tau aggregation	−	[48]
Aluminum chloride (alcl3-) induced Alzheimer's disease	Rat	Oral gavage	60 days	CA	200 mg/kg	Reduce the GFAP-positive cells	Reduce the progression of neurofibrillary degeneration, loss of dendritic spines, and appearance of neuritic plaques	Improve T-maze test	[44]
Presenilin 1/2 conditional double knockout mice	Mouse	IP	90 days	TCA	240 ppm	Suppress the NF-*κ*b signaling pathway	Prevent the upregulation of iNOS, COX-2, IL-1*β*, and TNF-*α* and rescue memory impairment and synaptic dysfunction	Improve novel object recognition test, Morris water maze, and Y-maze	[51]
Methamphetamine-induced spatial learning and memory deficits	Rat	IP	7 days	CA	80 mg/kg	Activate the ERK signaling pathway	Improve cognitive and learning functions	Improve memory impairment in Morris water maze	[50]
LPS	Mouse/primary microglia	IP/incubation	28 days	TCA	50 mg/kg/10 *μ*M	Inhibit MEK1/2-ERK1/2 signaling pathway	Decrease NO production and IL-1*β* release in primary microglia and improve memory impairment	Improve open field, novel object recognition task, and Morris water maze test	[53]
LPS	Mouse	IP	7 days	TCA	50 mg/kg/day	Inhibit the NF-*κ*B activation and attenuate the level of IL1*β*, MDA, and caspase-3	Modulate Nrf2 antioxidant defense in the hippocampus, inhibit neuroinflammation, apoptosis, and amyloid protein burden	Improve spatial and nonspatial memories impairment in Morris water maze and object recognition test	[52]
MPTP/MPP^+^-induced neuronal cell injury	Mouse/human neuroblastoma BE(2)-M17 cells	IP	7 days	CA	10 mg/kg	Upregulate p62 and reduce the rate of autophagy	Inhibit autophagy	−	[66]
6-OHDA	PC12 cell lines	Incubation	24 h	CA	10 *μ*M	−	Increase viability of 6-OHDA-treated cells and decrease the ROS generation	−	[65]

AD: Alzheimer's disease; PD: Parkinson disease; CA: cinnamaldehyde; MPTP: 1-methyl-4-phenyl-1,2,3,6-tetrahydropyridine, MPP+: 1-methyl-4 phenylpyridinium; Nrf2: nuclear factor erythroid 2-related factor 2; ROS: reactive oxygen species; 6-OHDA: 6-hydroxydopamine; IP: intraperitoneal; COX2: cyclo-oxygenase 2; IL-1*β*: interleukin-1*β*; TNF-*α*: tumor necrosis factor-*α*.
